# Range of detection and naturalistic search performance for spotted lanternfly (*Lycorma delicatula*) egg masses

**DOI:** 10.7717/peerj.21387

**Published:** 2026-06-09

**Authors:** Sally Dickinson, Mizuho Nita, Edgar O. Aviles-Rosa, Nathan Hall, Erica N. Feuerbacher

**Affiliations:** 1 School of Animal Sciences, Virginia Polytechnic Institute and State University (Virginia Tech), Blacksburg, VA, United States of America; 2 AREC, Virginia Polytechnic Institute and State University (Virginia Tech), Winchester, VA, United States of America; 3 Animal and Food Sciences, Texas Tech University, Lubbock, TX, United States of America

**Keywords:** Spotted Lanternfly, Conservation Detection Dogs, Participatory Science, Detection dog evaluation measures

## Abstract

The spotted lanternfly (*Lycorma delicatula*, SLF) poses a significant threat to U.S. agriculture, particularly vineyards, hops, and ornamental plants. Early detection of SLF egg masses is critical for limiting spread, yet current strategies are constrained by the availability of trained personnel and are extremely time-consuming. Detection dogs have shown strong potential for locating SLF egg masses with high accuracy, and training can be completed using devitalized samples, eliminating the risk of accidental release of this invasive insect. In a prior study, we demonstrated that participatory science teams, volunteer handlers with scent detection experience, could successfully train their companion dogs to detect devitalized SLF egg masses. This follow-up study evaluated whether selected teams from the original cohorts could perform under more complex, operationally relevant conditions. Specifically, we assessed detection accuracy and field performance across two experimental settings: (1) a range of detection (RoD) tests to estimate reliable detection distance, and (2) a naturalistic field search in previously unsurveyed areas with unknown target presence, allowing comparison with human surveyors. In the RoD trials, dogs demonstrated the highest sensitivity (0.52) at 0–5 m, declining to 0.06 at 10–15 m, with overall precision ranging from 0.61 to 0.92 across distance bands where detections occurred. Several dogs also successfully generalized from devitalized training aids to naturally occurring, previously undetected SLF egg masses. In naturalistic searches, canine teams located more confirmed SLF egg mass sites than trained human searchers, highlighting their ability to detect cryptic targets under real-world conditions. Although not all canine alerts could be confirmed, the results indicate that trained community detection teams can effectively complement or enhance traditional survey methods. Overall, these findings support the operational feasibility of participatory science detection teams for SLF surveillance. Despite range limitations, trained community dog–handler teams can successfully detect SLF egg masses and, in some cases, outperform human searchers, offering a scalable, biosecure, and cost-effective approach to invasive species detection.

## Introduction

The spotted lanternfly, *Lycorma delicatula White* (Hemiptera:Fulgoridae) (SLF) is an invasive planthopper that has rapidly spread across the eastern United States since its introduction in 2014 (Urban, 2020). This insect poses a critical threat to a range of agricultural crops, most notably grapevines, where infestations can cause significant yield losses and increased production costs (Urban, 2020; Dara, Barringer & Arthurs, 2015). Targeting the egg mass stage of the SLF life cycle offers a strategic advantage, as each mass contains 30–60 eggs and can be detected and removed before hatching, interrupting population growth and long-distance spread (Urban, 2020). However, locating egg masses in the field presents a considerable challenge. SLF egg masses are considered cryptic, meaning their physical characteristics and placement reduce the likelihood of detection by predators, or, in this case, human surveyors. Each egg mass contains eggs laid in rows and covered by a protective secretion. This coating, produced by the female SLF, is initially white and glossy but dries to a gray-brown, putty-like layer that cracks and weathers over time, closely resembling a smear of dried mud or lichen (Dara, Barringer & Arthurs, 2015). This resemblance allows the egg masses to blend into common substrates such as bark, rocks, or outdoor equipment, making visual detection particularly challenging (Urban, 2020).

Conservation Detection Dogs (CDD) have emerged as an efficient and accurate method for locating SLF egg masses (Keller & Hoover, 2023), particularly in contexts where human visual surveys are limited by time and access to hidden surfaces, for example, crevasses in tree limbs or internal spaces of packaged materials. Prior work has demonstrated that trained dogs can successfully detect both devitalized (Aviles-Rosa et al., 2023; Essler et al., 2021) and live SLF egg masses (Dickinson et al., 2025) and outperform humans in structured search tasks (Fuller et al., 2024). However, traditional conservation detection programs face scalability limitations due to the financial and logistical burden of procuring, training, and maintaining working dog teams (Rutter et al., 2022). As the geographic extent of SLF infestation continues to expand (Fuller et al., 2024; Bullock, Moylett & Para, 2017; Essler et al., 2021), the availability of professional canine detection teams remains insufficient to meet operational demand.

To address this gap, our previous study (Dickinson et al., 2025) evaluated whether companion dogs trained by experienced sport detection handlers could contribute meaningfully to SLF detection efforts. Using a participatory science model, we recruited community scientist teams and provided them with devitalized SLF egg training aids. After completing a period of independent training with oversight from local trainers, dogs were evaluated using a standardized odor recognition test (ORT) and field evaluation (FE). A subset of teams that successfully passed both evaluations were further assessed for their ability to transition detection behavior to live SLF egg masses. Results demonstrated a sensitivity of 0.82 in the ORT and 0.61 in the FE, with precision exceeding 0.87 in both settings. The teams evaluated on live SLF eggs demonstrated sensitivity of ≥0.80 following a brief acclimation period, further supporting the feasibility of using devitalized training aids for initial training. These performance metrics are comparable to those reported in other conservation detection dog (CDD) programs, where sensitivity values between 0.60 and 0.85 and high precision are typical for a variety of species, even among professionally handled teams operating under field conditions (Cristescu et al., 2015; Beebe, Howell & Bennett, 2016).

Although these results provide strong evidence that participatory science detection dog teams can meet baseline performance standards in controlled and semi-structured conditions, additional research is needed to understand how these teams perform under more complex and variable operational contexts. Real-world applications often involve diverse search environments, and less predictable odor presentations. The ability of participatory teams to apply their training and detection skills while maintaining detection accuracy under variable field conditions is critical for assessing their potential utility in agricultural biosecurity programs.

The present study builds upon our initial findings by evaluating the participatory science dog teams’ detection performance in more applied search scenarios. Using a subset of dog-handler teams who completed the field evaluation in our original study. We conducted two follow-up assessments. The first was a range of detection test, designed to evaluate each team’s ability to detect SLF egg masses at varying distances and establish operational detection ranges. The second was a naturalistic search in a historically infested area, intended to simulate real-world deployment conditions and assess how teams performed in an unstructured environment with unknown target presence. Together, these experiments aimed to further refine the operational strengths and limitations of participatory detection teams and inform future strategies for integrating community science models into invasive species management frameworks.

## Study Overview

This follow-up study engaged a subset of dog-handler teams from our prior work (Dickinson et al., 2025), specifically, those that had completed the field evaluation (FE) phase and demonstrated successful field search performance. All participating dogs had been previously trained using devitalized SLF egg training aids (SLF-TA), which were constructed from field-collected egg masses rendered non-viable through storage at a minimum of −80 °C for 48 h and sealed in stainless steel mesh packets to ensure biosecurity and odor consistency. For a full explanation of the materials and methods used, see Dickinson et al. (2025). The present study consisted of two experimental components: (1) a range of detection (RoD) test and (2) a naturalistic field search. One group included dogs that participated in both components, dogs at all other sites participated in only one.

1. **Experiment 1—range of detection:** This component assessed the operational detection range of dogs trained on SLF egg masses. Each team was evaluated on its ability to locate a single target sample (devitalized or live), with distances varied systematically to record the closest miss and farthest accurate detection.
2. **Experiment 2—naturalistic search effectiveness:** This component evaluated team performance in a real-world search scenario. The search took place in a designated, outdoor area that remained uncontrolled for SLF egg masses. The presence, number, and locations of SLF egg masses were unknown to both handlers and researchers at the time of the search. Teams operated as they would during an operational deployment, conducting independent searches and alerting to suspected targets. All alerts were subsequently verified through direct inspection by an experimenter. To provide a comparison, trained human searchers also surveyed the area visually (prior to the dogs’ search), allowing for an assessment of canine team effectiveness relative to standard human-based detection methods.

The materials, methods, and results for each experiment are presented separately. A general discussion integrating findings from both experiments follows at the end. Approval to conduct these studies was granted by the Virginia Tech Division of Scholarly Integrity and Research Compliance, Institutional Animal Care and Use Committee (IACUC #23-025).

## Experiment 1—Range of Detection

A critical factor in determining how to effectively deploy a CDD is understanding the team’s operational range of detection (RoD). Odor dispersal in the environment is influenced by the physical and chemical properties of the odor source (McKeague, Finlay & Rooney, 2024; Bennett, Hauser & Moore, 2020), meteorological conditions such as wind speed, humidity, and temperature gradients, (Wohlfahrt et al., 2023) and terrain features like vegetation density, elevation changes, or barriers (Fuller et al., 2024). While all odors have a finite realistic detection range, establishing RoD values allows for more precise search planning, improves deployment efficiency, and informs expectations about detection performance in field conditions. For example, if the anticipated RoD is 10 m under moderate wind conditions (8–13 km/h), grid patterns can be adjusted so that search lanes are spaced accordingly to ensure the desired search coverage is achieved. In contrast, if RoD drops to three m in dense vegetation or during still air conditions, search lanes may need to be narrowed or searches may need to be repeated from multiple angles to maintain coverage. Most importantly, RoD provides a basis for adjusting the search strategy to match the operational objective. In some cases, such as population density surveys or containment/reduction efforts, searches may require near-complete detection of all targets. In others, such as early detection or presence/absence surveys, it may be sufficient to detect a small number of targets to confirm infestation (Bennett, Hauser & Moore, 2020). Understanding the RoD allows handlers and planners to calibrate lane spacing, time spent, and team configuration to meet these varying goals effectively and efficiently.

This experiment was designed to evaluate the range of detection for dogs trained on devitalized SLF egg mass odor, using either devitalized or live egg samples. Specifically, we aimed to identify two performance parameters for each dog: the farthest distance at which a dog could successfully detect and alert to a target (farthest hit), and the closest distance at which the dog failed to detect a present target (closest miss). These metrics provide a practical framework for understanding both individual variability in detection capacity and overall limitations in scent accessibility for SLF egg mass targets.

### Methods and materials

#### Participants

Participants in Experiment 1 included 26 dog-handler teams from the original study Dickinson et al. (2025), who had successfully completed both the Odor Recognition Test (ORT) and Field Evaluation (FE), and who were available for testing at one of four designated locations in Ohio, Pennsylvania, Maryland, and Virginia.

#### Setting

**Figure 1:**
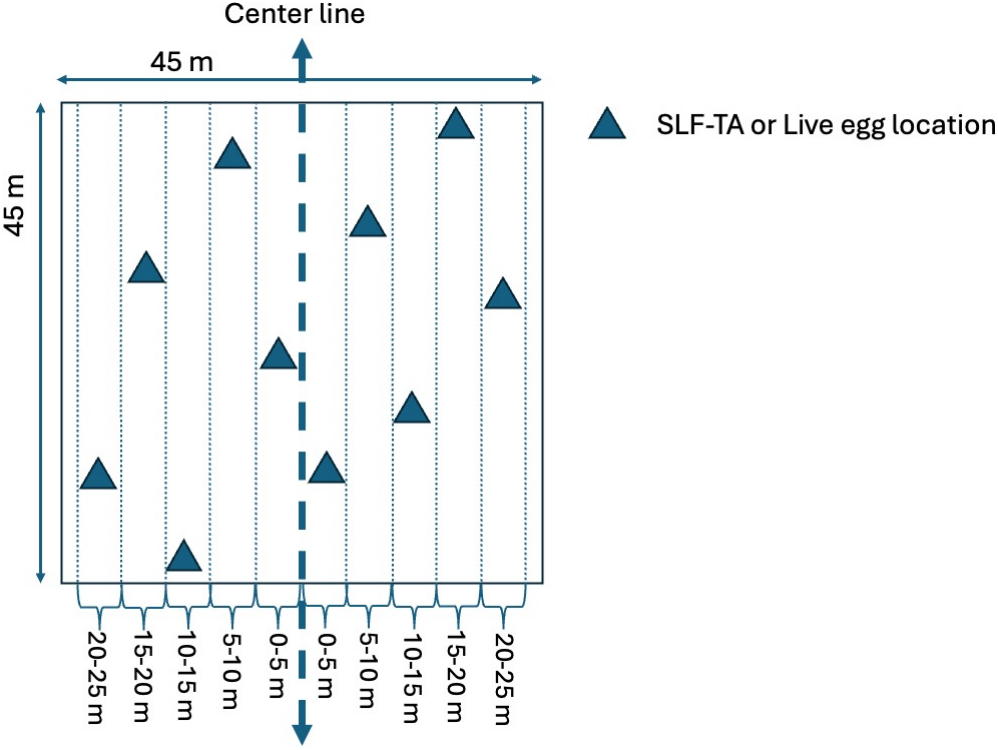
Example of placement of SLF-TA or live SLF eggs for the Range of Detection experiment.

The RoD experiments were conducted at a variety of site types, including wineries, wooded areas, and community parks. Each location was first visually inspected for safety hazards and for the presence of SLF egg masses. Only areas where no visible egg masses were detected were used for testing. At each site, we designated a 45 m × 45 m test area using flagging tape. We selected areas based on habitat features commonly associated with SLF egg mass deposition, such as tree trunks, vine posts, stacked lumber, and other vertical or semi-sheltered surfaces. A centerline was marked along the center of the area to serve as the path along which targets were placed (see Fig. 1, and for details on the target placement). In vineyard settings, the centerline was typically positioned between adjacent vine rows, while in wooded or park environments, it was placed along an existing walking path or other accessible corridor through the terrain.

#### Evaluators

For each RoD trial a specific person was designated as the evaluator and was responsible for setting up and recording the teams’ responses in the area. During the experiment, the evaluator remained neutral and did not interact with the handler. The evaluator observed each search and recorded the location at which the dog deviated from the center search line, whether an alert was given, and the object or location to which the dog alerted.

#### Odors

The RoD experiment used either SLF-TA (Spotted Lanternfly Training Aid; devitalized egg masses) or live SLF eggs as target odors. The choice of target type for each dog was determined by two factors: seasonal availability, live SLF eggs were only accessible during the winter and early spring months, and whether the dog had participated in the Live Egg Odor Recognition Test (LE-ORT). The LE-ORT, which was conducted immediately prior to the RoD trials when live samples were used, served to ensure that each dog had demonstrated to be trained to alert to live SLF egg masses before participating in a live-target RoD trial. Consequently, if live eggs were available at a given location, only dogs that had successfully completed the LE-ORT were assigned to the live condition; dogs that had not completed the LE-ORT were assigned to the devitalized condition, regardless of live egg availability. All dogs at a given testing location were assigned the same target type to avoid cross-contamination and maintain consistency in odor exposure. In trials where live SLF eggs were used, we harvested the eggs by pruning a short piece of branch or slicing off the bark to which the eggs were attached, allowing the mass to remain intact and adhered to its original substrate. For devitalized targets (SLF-TA), we followed the protocol described in our prior study (Dickinson et al., 2025): field-collected egg masses were rendered non-viable through −80 °C storage and sealed in stainless steel mesh packets. This packaging ensured biosecurity and odor consistency.

We placed a total of 10 targets (SLF-TA or live SLF eggs) at incremental distances from the designated center line of the search area, with targets placed on each side of the centerline at five distance intervals: 0–5 m, 5–10 m, 10–15 m, 15–20 m, and 20–25 m. As such, there was a target placed at each distance on each side of the center line. Targets were placed in a randomized order for each trial to prevent the team from detecting a pattern and to reduce the influence of pre-existing side bias in the dog on detection outcomes. The maximum distance assessed was 25 m from the centerline. Targets were concealed to ensure they were not visible to the handler or dog and were placed at a range of heights between 0 and 3 m above ground level. Target heights ranged from 0 to 3 m above ground level, consistent with the range of heights used during the Field Evaluation in our prior study (Dickinson et al., 2025), ensuring that vertical placement was within a range dogs had previously demonstrated the ability to detect. Each target location was recorded on a sketch map (see Fig. 1), which was also used to document team performance during the trial.

#### Procedures

On the day of the Range of Detection (RoD) experiment, each team arrived at a pre-designated time. Handlers were instructed to maintain their dogs’ typical routine, as they would when preparing for a scent detection event. At the assigned time, the evaluator oriented the handler (without the dog present) to the search area, identifying the boundaries and the designated center search line. Each trial consisted of a single pass along the center line in one direction only; handlers were not permitted to walk back and forth. Teams had 10 min to complete a trial, defined as a continuous search from one end of the center line to the other. Dogs could work on- or off-leash, but both the handler and dog were required to generally remain on the center line to standardize exposure to target odors. If the dog deviated laterally from the line to investigate or alert, the handler was permitted to follow. However, after evaluating the behavior (*e.g.*, confirming an alert), the team was instructed to return to the center line and continue moving in the original direction of travel. Each team was encouraged to complete at least two trials, starting from opposite ends of the center line, to help control for environmental variables such as wind direction or terrain gradient that could influence odor dispersal. These passes were not independent replicates; targets remained in place between passes and teams simply traversed the centerline in the opposite direction. All trials for a given dog were conducted on the same day, although they were not required to be performed consecutively; handlers were allowed to rest their dogs between trials and proceed at a pace appropriate for their dog’s condition and readiness. If a team wished, or if the dog was able to complete more than two trials, they were assigned to a different RoD setup with a new target layout. Each dog was permitted to complete only two trials per RoD configuration: one in each direction along the center line.

#### Measurement

As the handler and dog moved along the center search line, the evaluator recorded the locations where the handler identified that the dog was exhibiting behavior consistent with detecting the target odor and noted which target the dog alerted to. The handlers verbally called out alert locations during the trial, and the evaluator documented those locations. The evaluator provided no feedback during the RoD experiment. Handlers were permitted to deliver reinforcement at their discretion when they believed an alert was accurate. Detection in this study was a combined effort between dog and handler: the dog was responsible for detecting and communicating the presence of odor, and the handler was responsible for correctly interpreting the dog’s behavior. We used a standard confusion matrix (Wickens, 2002) to classify the responses and calculate sensitivity and precision (Table 1, Eq. (1)). Sensitivity indicates, of all the real targets, how many were found. Precision tells us, of all alerts, how many were correct.

**Table 1:** Standard confusion matrix for classification of responses.

	**Target present**	**Target absent**
**Target detected**	True positive (TP)	False positive (FP)
**Target Not Detected**	False Negative (FN)	Correct Rejection (CR)

(1) \begin{eqnarray*}\mathrm{Sensitivity}= \frac{\mathrm{TP}}{\mathrm{TP}+\mathrm{FN}}  \mathrm{Precision}= \frac{\mathrm{TP}}{\mathrm{TP}+\mathrm{FP}}  \end{eqnarray*}

#### Analysis

Detection performance was analyzed using a generalized linear mixed-effects model (GLMM) with a binomial error distribution and logit link. The response variable was the number of successful detections (true positives) out of the total number of detection opportunities (true positives + false negatives) per dog per distance band per trial. Distance band and stimulus type (live *vs.* devitalized) were included as fixed effects, and dog-handler team ID was included as a random intercept to account for the non-independence of observations within teams across trials. Distance was treated as an ordered predictor, and inference focused on the linear trend across distance bands. Results were pooled across the four testing sites (Ohio, Pennsylvania, Maryland, and Virginia); site was not included as a random effect due to the limited number of observations per site. The multi-site design was intentional, as our goal was to evaluate detection performance across a range of naturalistic field conditions representative of real-world deployment contexts. Models were fitted in R (version 4.5.2) (R Core Team, 2025) using the lme4 package (Bates et al., 2015).

## Results

A total of 39 RoD trials were completed by 26 individual dog-handler teams. Eighteen handlers completed a single trial, citing factors including dog age or condition, environmental conditions, task difficulty, and physical challenges associated with the terrain. Of the 39 trials, 25 used live egg samples and 14 used devitalized egg samples. No samples, live or devitalized, were detected beyond 15 m from the center search line. Table 2 summarizes pooled sensitivity and precision across distance bands. Distance bands beyond 15 m are not shown because no detections occurred at these distances, resulting in zero sensitivity and undefined precision.

**Table 2:** Descriptive sensitivity and precision for live and devitalized SLF egg targets across distance bands. Values represent pooled proportions across all trials. Distance bands beyond 15 m are not shown because no targets were detected at these distances, resulting in zero sensitivity and undefined precision.

	0–5 meters		5–10 meters		10–15 meters	
	Sensitivity	Precision	Sensitivity	Precision	Sensitivity	Precision
Live SLF eggs	0.52	0.59	0.25	0.5	0.03	0.83
Devitalized eggs	.51	0.82	0.31	0.72	0.1	1
Average	0.52	0.70	0.28	0.61	0.06	0.92

**Figure 2:**
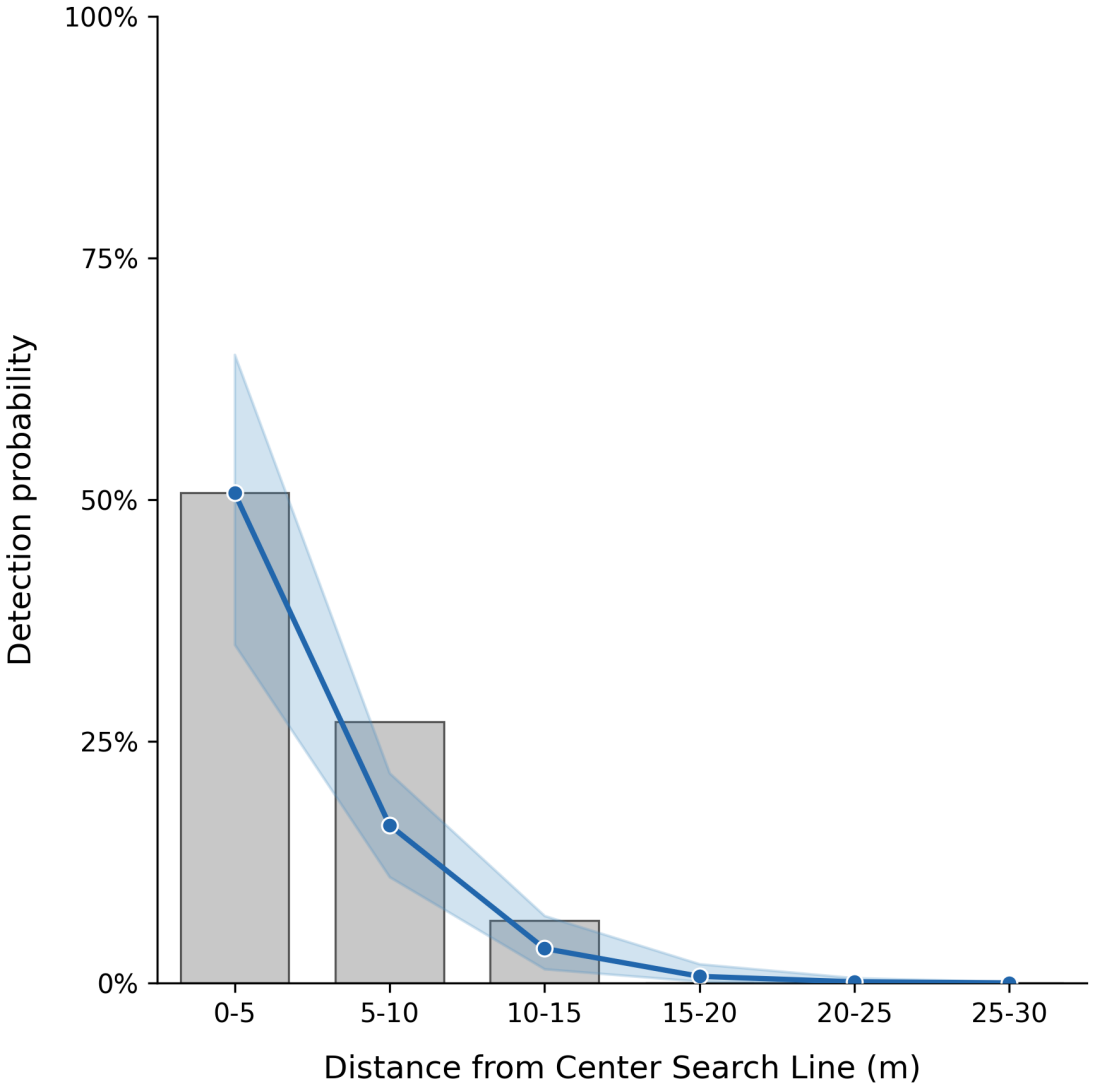
Bars represent detection proportions across stimulus type. Points and solid line indicate model-predicted detection probabilities from a generalized linear mixed-effects model, with shaded bands representing 95% confidence intervals. Detection probability declined significantly with increasing distance from the search line (*P* < .001).

Detection probability declined significantly with increasing distance from the search line (*χ*^2^ = 154.47, df = 1, *P* < .001), while stimulus type had no effect on detection (*P* = .90). There was no evidence of a distance × stimulus interaction (*P* = .93). When distance was modeled as an ordered linear trend, the odds of detection decreased by approximately 81% with each successive distance band (odds ratio =0.19, 95% CI [0.12–0.27]) (Fig. 2).

**Figure 3:**
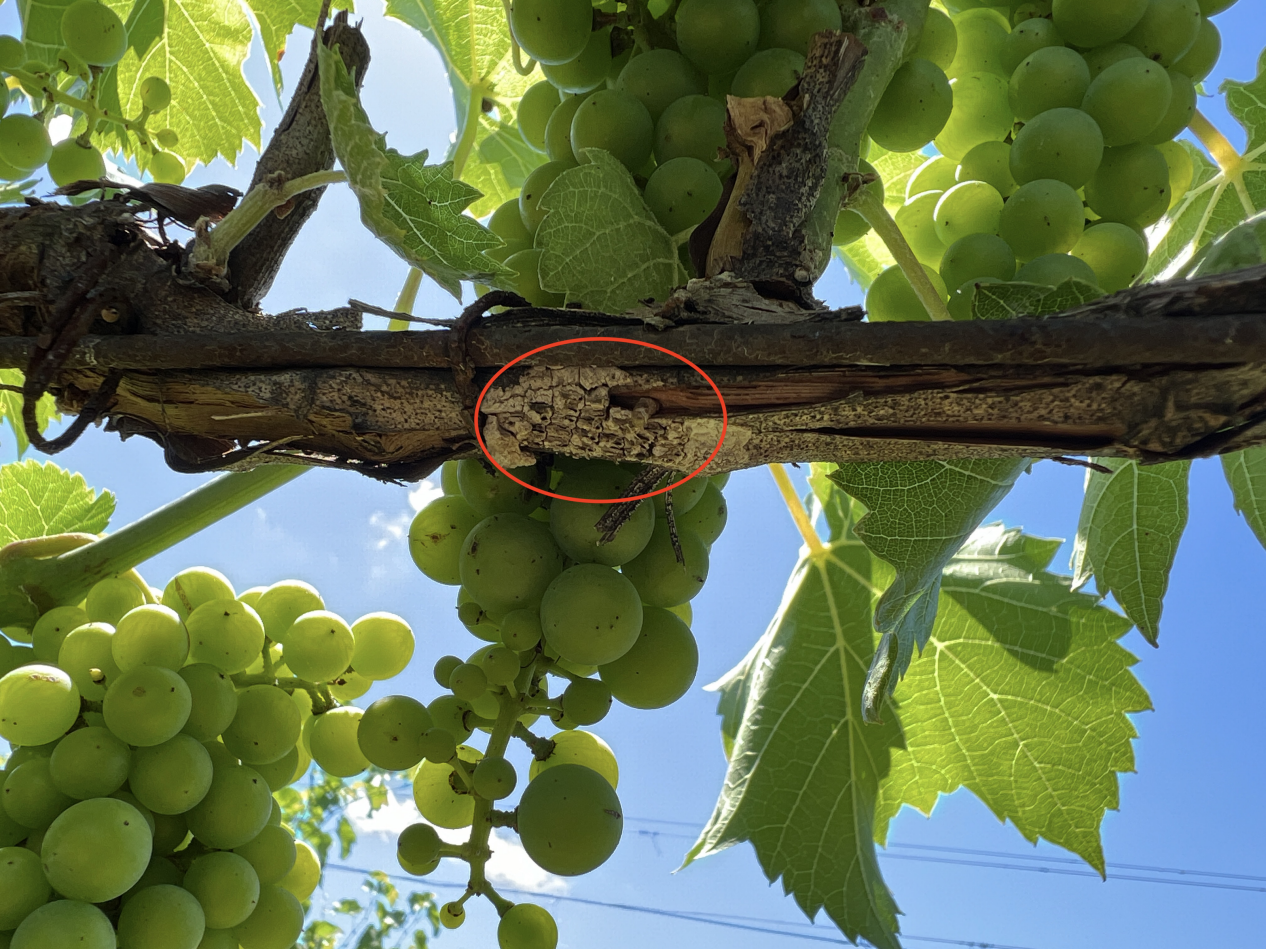
Naturally occurring SLF egg mass. Photograph shows a naturally occurring egg mass (red circle) located by a dog–handler team during the RoD experiment. The SLF egg mass is on the underside of a grapevine cordon (the horizontal, perennial part of the vine). Photo credit: Sally Dickinson.

During seven trials at one of the RoD site, four dog-handler teams alerted to seven locations where no experimental targets (SLF-TA or live egg masses) had been placed. After completion of the trials, those locations were visually inspected and naturally occurring SLF egg masses were confirmed at each site. Figure 3 shows one of the egg masses found by the dogs. These naturally occurring egg masses were not included in the quantitative analyses of sensitivity and precision (Table 2, Fig. 2) because their presence and total number were unknown prior to testing, and thus could not be used to calculate false positives or false negatives. Of the seven confirmed alerts, five were located within 0–5 m from the center search line and two were between 5–10 m. Each alert occurred during a trial in which the dog was the first to search that particular transect layout.

## Experiment 2—Naturalistic Search Effectiveness

To evaluate the training and evaluation process under real-world conditions, we asked participants to search areas that had not been previously surveyed. Both trained dog-handler teams and experienced human searchers participated in this component, allowing for direct comparison of their search outcomes. The experiment was conducted during the winter months, when SLF egg masses are present in the environment. The primary objective was to assess the relative effectiveness of searches conducted by trained human searchers *versus* those conducted by trained participatory science dog teams.

### Methods and materials

#### Participants

Experiment 2—Naturalistic Search Effectiveness, included two participant groups: (1) nine dog-handler teams from the original study who had successfully completed the Odor Recognition Test (ORT), Field Evaluation (FE), and Live Egg ORT (LE-ORT), and were available for follow-up testing; and (2) five experienced human searchers with prior training in identifying SLF egg masses. Two human searchers participated at Location 1 and three at Location 2. The human participants had previously participated in SLF egg mass detection as part of population density surveys and eradication programs, providing them with relevant field experience in recognizing and locating egg masses under naturalistic conditions.

#### Setting

We conducted the search effectiveness experiment at two locations. At location 1, we selected two, 2,023 square meters (1/2 acre) site of managed woodland with several species of mature trees, limited underbrush and some dead trees on the ground. The area was part of an estate consisting of woodland, grassland and outbuildings. All five dog-handler teams searched both sites, with a break between searches. At location 2, we selected one 2,023 square meters (1/2 acre) site that had several species of mature trees growing, limited underbrush, and some dead trees on the ground. The area was considered waste land between a housing development and a county yard waste collection site. All four dogs searched the area. All areas we surveyed the area for hazards and clearly marked the corners of the search area with flagging tape.

#### Evaluators

On the day of the Naturalistic Search Effectiveness experiment, dog-handler teams and human searchers arrived simultaneously. Dog handlers were instructed to maintain their dogs’ typical pre-search routines, as they would prior to a standard scent detection event. A joint briefing was provided to all participants, outlining the general search area, its dimensions, and procedural expectations. Both dog handlers and human visual searchers were instructed to notify the accompanying researcher immediately upon locating a suspected SLF egg mass, so the location could be marked using GPS. A single evaluator, a member of the research team, observed all searches and recorded the locations of all identified SLF egg masses using a handheld Garmin GPS Alpha 300 (Garmin, Lenexa, KS, USA).

The order of searches was fixed: at both test locations, human visual searchers conducted their searches first, followed by dog-handler teams, one at a time. No information about prior finds was shared with subsequent teams to avoid bias or unintentional cueing. Each search, whether conducted by a dog-handler team or a human searcher, was limited to 10 min. Participants were instructed to continue searching for the full duration, even after making one or more finds. Search strategies were left to the discretion of the participants. Dog handlers were permitted to work their dogs using their preferred handling style, on- or off-leash, and provide reinforcement as they normally would.

#### Measurement

To record alert locations, we overlaid a grid onto an aerial photograph of the site obtained from Google Maps. The grid was georeferenced using four GPS points positioned at the corners of the 45 m × 45 m search area and divided the space into 10 × 10 cells, each approximately 4.5 m × 4.5 m. Alert locations were marked using the GPS coordinates corresponding to either the dog-handler team’s alert or the point identified by the visual searcher. Upon completion of all the searches, researchers and visual searchers revisited each alert location to identify and count all SLF egg masses located within a 3-meter radius, measured both horizontally and vertically, from the point of indication.

## Results

**Figure 4:**
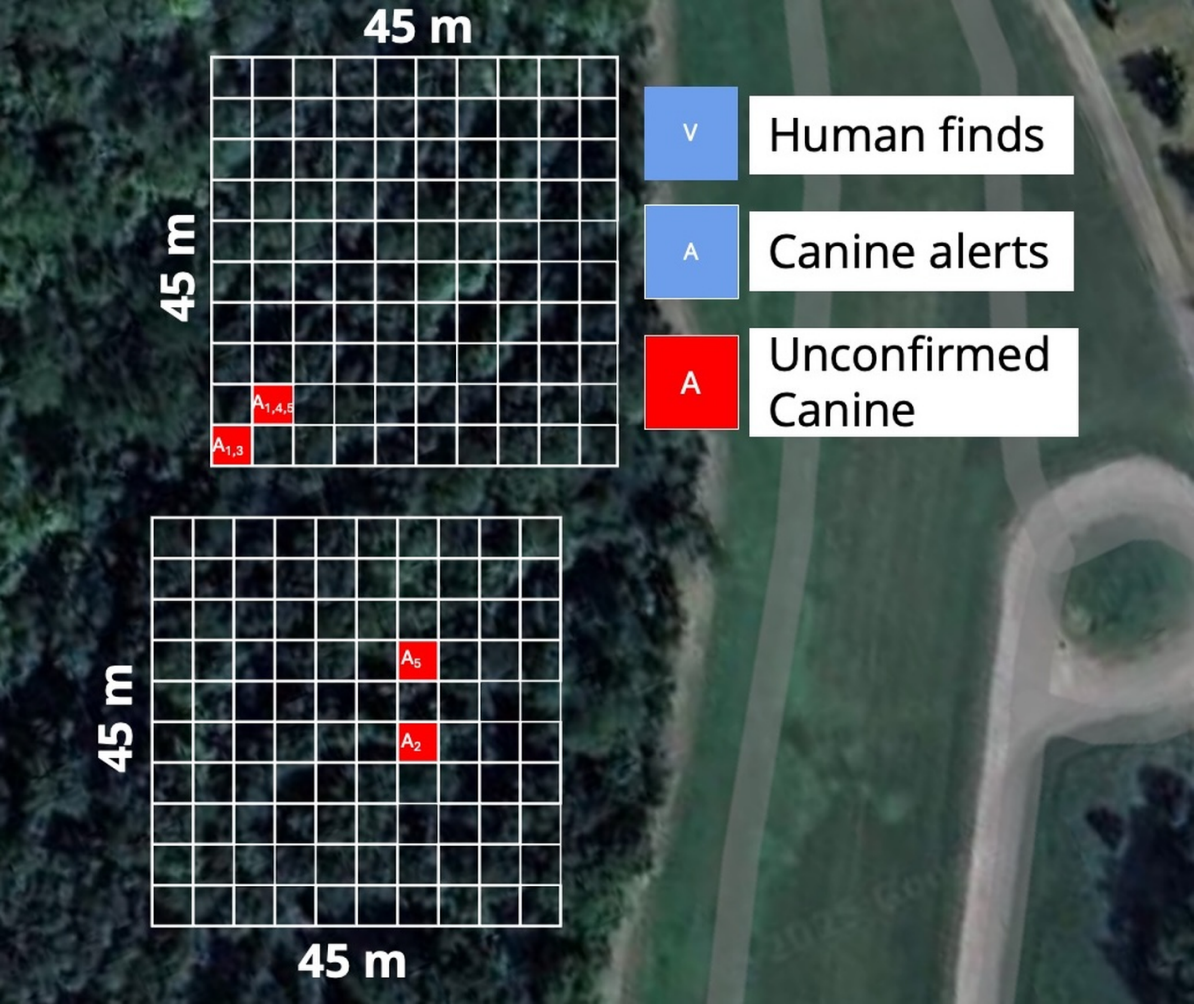
Location 1—naturalistic search effectiveness map. Satellite image with a 10 × 10 grid overlay showing canine alert locations during the naturalistic search. Alerts from dog–handler teams are marked with “A” and a subscript number indicating the dog(s) that alerted at each location (*e.g.*, ***A*_2,5_** denotes alerts from dog numbers 2 and 5). All alert areas are shown in red, as no SLF egg masses were confirmed at any location. Base map imagery ©Google Maps (accessed August 2025).

**Figure 5:**
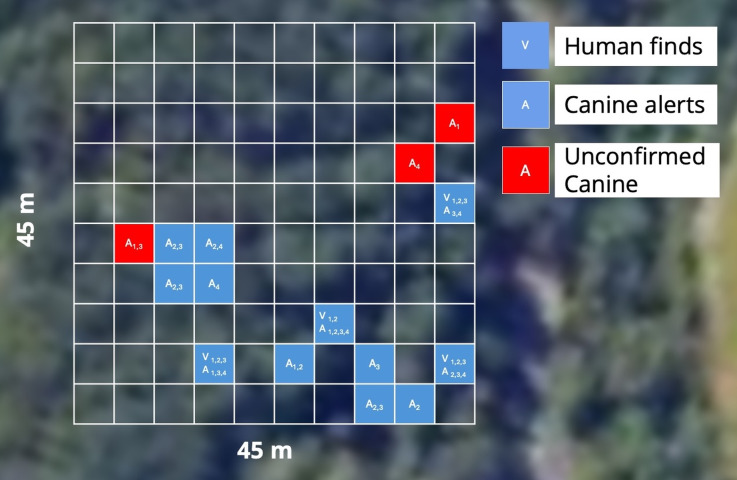
Location 2—Naturalistic Search Effectiveness map. Satellite image with a 10 × 10 grid overlay showing detection events from both dog–handler teams and human searchers during the Location 2 naturalistic search. Alerts from dog teams are marked with “A” and a subscript number indicating the dog(s) that alerted at each location (*e.g.*, ***A*_1,4_** denotes alerts from dog numbers 1 and 4). Human searcher detections are denoted by **V**. Areas colored blue represent locations confirmed to contain at least one SLF egg mass, while areas colored red indicate unconfirmed canine alerts. Base map imagery ©Google Maps (accessed August 2025).

We report the results for the five teams at the separate areas in Location 1 (Fig. 4) and the four teams in Location 2 (Fig. 5), as well as the aggregate totals from both sites (Table 3).

**Table 3:** Naturalistic search effectiveness. Table showing number of confirmed and unconfirmed alert each dog and human made, by site.

**Human/Dog**	**Visual finds**	**Confirmed dog finds**	**Unconfirmed dog alerts**
Location 1, Area 1			
Human 1	0	N/A	N/A
Human 2	0	N/A	N/A
Dog 1	N/A	0	2
Dog 2	N/A	0	0
Dog 3	N/A	0	1
Dog 4	N/A	0	1
Dog 5	N/A	0	1
Location 1, Area 2			
Human 1	0	N/A	N/A
Human 2	0	N/A	N/A
Dog 1	N/A	0	0
Dog 2	N/A	0	1
Dog 3	N/A	0	0
Dog 4	N/A	0	0
Dog 5	N/A	0	1
Location 2			
Human 1	3	N/A	N/A
Human 2	3	N/A	N/A
Human 3	2	N/A	N/A
Dog 1	N/A	3	2
Dog 2	N/A	8	0
Dog 3	N/A	8	1
Dog 4	N/A	6	1

### Location 1 results

Figure 4 shows the site map with the grid overlay and locations of the canine alerts. The two human searchers did not locate any SLF egg masses during the search, while the dog teams alerted to four areas. Despite thorough inspection by trained human searchers, including checks higher in trees, in fallen timber, and other likely locations, no egg masses were confirmed at those alert sites.

### Location 2 results

Figure 5 shows the map with the grid overlay and the locations of the human finds and the dog alerts. The three human searchers found SLF egg masses in four locations. The dog teams alerted to these four locations and a total of eight confirmed additional locations. Three additional locations remained unconfirmed as having SLF egg masses after inspection. Although the total number of naturally occurring SLF egg masses in this area could not be determined, the pattern of alerts provides shows agreement between dogs. Of the locations where dogs alerted, eight sites were independently confirmed by more than one dog handler team, as shown in Fig. 5. Several of these sites were alerted to by two or more dogs (*e.g.*, *A*_1,4_), indicating consistent responses across teams at those positions. In contrast, three locations were alerted to by only a single dog and remained unconfirmed after inspection, suggesting either isolated false alerts or egg masses that were missed visually. Overall, the majority of confirmed alerts were located by multiple dogs, demonstrating substantial agreement among teams despite the unknown total number of egg masses.

## Discussion

We evaluated the field performance of participatory science dog-handler teams who had previously completed standardized training and passed the Odor Recognition Test (ORT) and Field Evaluation (FE) described in our earlier study (Dickinson et al., 2025); a subset of these teams also completed a Live Egg ORT (LE-ORT) prior to participating in the current experiments. In the current study, we assessed teams under two operationally-relevant scenarios: (1) Range of Detection experiment and (2) Naturalistic Search Effectiveness. In the Range of Detection test, the teams were more successful at locating targets positioned closer to the search path, with sensitivity highest (0.52) at 0–5 m and declining with distance. In the Naturalistic Search Effectiveness experiment, canine teams outperformed trained human searchers, detecting up to three times as many confirmed SLF egg mass locations in some field conditions. These results underscore the capacity of trained dog-handler teams to detect cryptic targets under operational constraints and highlight their value as a complementary tool in invasive species surveillance.

### Range of detection

The RoD results demonstrated that SLF-trained detection dogs had a sensitivity of 0.52 at 0–5 m, which declined to 0.28 at 5–10 m and just 0.06 at 10–15 m. No targets were detected beyond 15 m. Precision, by contrast, remained relatively high at all distances where detections were made, reaching 0.92 overall at the farthest range. The higher precision observed at 10–15 m relative to 5–10 m is likely a mathematical artifact of the very small number of alerts at that distance; when few alerts are made, even a single false positive has a proportionally large effect on precision, and this value should therefore be interpreted cautiously. By evaluating both sensitivity (how many targets were found of the total) and precision (of the alerts, which were correct), we captured not only the dogs’ ability to detect targets in the environment, but also their accuracy when they did respond. From an operational standpoint, these results suggest that search grid spacing should be less than 10 m to maximize the probability of detection in field deployments.

During the Range of Detection trials, dogs worked with their handlers under conditions typical of field deployments, with handlers blinded to target placement. Most participating dogs (25 of 26; 96%) were trained primarily in nosework-style detection tasks, which emphasize close, methodical searching in proximity to the handler. As a result, detections at closer distances may partially reflect the dogs prior experience of how far away from their handler they will locate targets. Such learning is an inherent of all detection dog training and is reflected in how canine team operates under real-world conditions. Notably, the dog that located targets at greater distances, including the furthest naturally occurring SLF egg mass detected during the RoD study, had prior training as a cadaver detection dog, a discipline that often emphasizes independent searching and longer working distances from the handler. These findings align with recommendations for other conservation detection dog (CDD) applications, where effective search widths commonly fall within the 5–10 m range, depending on odor characteristics and terrain complexity (McKeague, Finlay & Rooney, 2024). This is consistent with broader CDD benchmarks; Glen & Veltman (2018) report that available data suggest most target taxa are detected by professionally trained detection dogs from an average of around 10 m, and recommend assuming this as a working detection distance in the absence of target-specific data. That our community science teams achieved reliable detection primarily within 0–10 m therefore aligns with established CDD performance benchmarks, while also reflecting the nosework-style training background of most participating dogs. However, direct comparison of our results with other SLF detection dog studies remains challenging due to variation in experimental design and reporting. For example, Fuller et al. (2024) quantified canine detection performance using a different methodological framework, limiting direct comparison with our results—a challenge also noted by McKeague, Finlay & Rooney (2024) in their review of methodological inconsistencies across CDD research. To address this issue, Bennett, Hauser & Moore (2020) recommended that CDD studies report both sensitivity and precision to enhance transparency, comparability, and operational relevance. By including these metrics, our findings contribute to a growing body of standardized performance data for detection dogs in applied invasive species management. Although vertical detection range was not formally assessed in the present study, targets were placed at heights between 0 and three m above ground level, consistent with prior training and evaluation protocols (Dickinson et al., 2025). This is operationally relevant given that Keller & Hoover (2023) found that over 50% of naturally occurring SLF egg masses were deposited above three m, with this proportion increasing to over 85% in plots with higher tree basal area, placing a substantial portion of egg masses beyond easy reach for human surveyors. Notably, during the Naturalistic Search Effectiveness experiment, several canine teams successfully alerted to SLF egg masses located high in trees, providing preliminary evidence that trained dogs can detect targets across a meaningful vertical range under field conditions. Formal assessment of vertical detection range represents an important direction for future research.

A notable incidental finding during the RoD trials was the detection of seven naturally occurring SLF egg masses by dogs trained exclusively on devitalized targets. These egg masses were found in areas that had been visually inspected and presumed clear prior to the trials. Because the total number of naturally occurring egg masses in the area was unknown, these alerts were excluded from statistical analysis. However, their discovery is operationally meaningful: the dogs successfully identified cryptic, naturally occurring targets under field conditions without any prior exposure to live SLF eggs. In our prior study (Dickinson et al., 2025), dogs trained on devitalized samples demonstrated successful transition to live SLF egg detection, but this occurred after brief training exposure to live samples. In contrast, the present finding demonstrates spontaneous generalization from devitalized to live SLF egg masses with no additional training or exposure, thereby providing strong evidence for the ecological validity of devitalized training aids. These unprompted detections also highlight the potential for canine teams to identify targets missed during visual surveys, reinforcing their value in real-world deployment scenarios and early detection efforts.

### Naturalistic search effectiveness

At Location 2, when accounting for the different number of participants in each group, dog teams confirmed an average of 3.0 egg mass locations per team, compared to 1.33 per human searcher, approximately a 2.25 fold higher detection rate per searcher. Across both groups, since searches were conducted blind to prior finds, each confirmed location represents an independent detection. In contrast, at Location 1, human searchers found no targets and the dogs’ alerts could not be confirmed. It remains unclear whether the area was truly free of egg masses, indicating false positives, or whether the canine teams correctly identified targets that were missed by visual search. These outcomes suggest that detection dogs can enhance or complement human survey efforts, particularly in complex or cluttered environments where cryptic targets such as SLF egg masses are easily overlooked. Importantly, while sensitivity could not be calculated due to the unknown number of egg masses in the area, the practical value of canine-assisted detection was evident. The dogs consistently located targets that were missed by trained human searchers, underscoring their utility in augmenting traditional survey efforts.

### Operational implications and limitations

The consistent detection performance observed with both devitalized and live SLF egg masses in the RoD experiment supports the continued use of devitalized samples for training and assessment (Rutter et al., 2021; Dickinson et al., 2025). This finding has important biosecurity implications, as it enables training and evaluation without the risk of accidental pest introduction, and facilitates the development of scalable, field-relevant preparation protocols. Beyond training utility, these results carry operational significance: the ability to generate reliable sweep width estimates using devitalized targets allows for more precise and informed search planning. For example, knowing the average effective detection range, accounting for specific environmental conditions, weather, and individual dog performance, can guide searcher spacing, and area coverage expectations. This helps align resource allocation with search objectives, whether for presence/absence surveys or intensive eradication efforts. Additionally, the use of devitalized targets supports predictive modeling of detection probability and enhances deployment design, reinforcing the practical integration of canine teams into SLF detection programs while maintaining biosecurity safeguards.

One limitation of both experiments is the difficulty of verifying false negatives (not detecting the target when the target is present) and false positives (alerting to another odor when no target is present) under field conditions, particularly in the naturalistic search (Bennett, Hauser & Moore, 2020). Without a complete census of egg masses in the area, it is not possible to calculate sensitivity. Future studies using controlled placements and double-blind evaluation protocols could help mitigate this issue. Additionally, although results were pooled across four geographically distinct sites, site was not modeled explicitly due to insufficient observations per site; future studies with larger sample sizes could incorporate site as a random effect to better partition environmental variation. Additionally, the unequal number of human searchers and dog-handler teams across locations limits direct group comparisons; future studies should aim for equal group sizes to enable more rigorous statistical comparison of detection rates. Although cost-benefit considerations are an important factor in evaluating the practical utility of canine detection teams relative to human searchers, a formal cost-benefit analysis was beyond the scope of the present study and represents a valuable direction for future research.

We designed the Naturalistic Search Effectiveness experiment (Experiment 2) to evaluate detection performance in areas with unknown target presence to most closely simulate a real-word use-case. By deploying dogs in previously unsurveyed(“virgin”) areas and comparing their detections to those of human visual searchers or established population density estimation methods, this approach eliminates artificially introduced odor sources and offers an ecologically valid assessment of real-world detection capability. Our findings align with those reported by Fuller et al. (2024), who also observed that detection dogs located SLF egg masses missed by trained human searchers in natural field settings. In that study, the probability of canine teams outperforming human searchers was greater in forested environments than in vineyard settings, highlighting the context-dependent nature of canine-assisted detection effectiveness. This same pattern is reflected in the present results.

The Location 2 Naturalistic Search area consisted of denser a and varried vegetation, irregular terrain, and visual obstructions more comparable to the forested sites described in Fuller et al. (2024) where dogs showed a clear advantage. In this setting, our canine teams identified eight previously unknown egg mass locations, doubling the number found by human surveyors. In contrast, the Location 1 Naturalistic Search area was far more open and visually accessible, and neither dogs nor humans produced confirmed detections. The RoD results further support this interpretation by showing that detection probability dropped sharply with distance, suggesting that visual occlusion and substrate complexity play a substantial role in shaping real-world performance. Taken together, these findings suggest that canine-assisted surveys are particularly effective in visually complex environments, aligning with our results and previous studies (Cristescu et al., 2015; Fuller et al., 2024), whereas their relative advantage diminishes when targets are readily visible or the habitat provides little concealment.

Finally, we observed that detection performance varied notably across dog-handler teams, with some dogs consistently locating targets at greater distances while others were more limited in their effective range. While this paper does not focus on individual-level analysis, the range of observed variability suggests that characteristics of both the dog and handler may influence detection outcomes. Relevant factors may include individual characteristics of the dog, training history, and search style, as well as handler experience, search strategy, and the quality of the dog-handler relationship. While CDD programs often favor working breeds such as German Shepherds, Hall et al. (2015) demonstrated that breed may not reliably predict odor discrimination performance, suggesting that breed selection for scent detection work has been driven more by historical convention than empirical evidence. The wider range of breeds represented in community science teams, compared to professionally selected CDD programs, may contribute to the performance variability observed across teams in the present study; however, given that breed alone does not reliably predict odor discrimination performance, other individual and handler-level factors are likely more explanatory. The breed composition of the participating community science teams is reported in Dickinson et al. (2025). Identifying which individual and team-level variables correlate with higher performance could inform future team selection, training protocols, and deployment strategies, and represents a valuable direction for further investigation.

### Conclusion

These findings demonstrate that trained participatory science detection dog–handler teams can be effectively deployed for the detection of *Lycorma delicatula* egg masses under operational field conditions. Although detection probability declined with increasing distance from the search path, dogs consistently detected targets within operationally relevant ranges (*i.e.,* within 15 m of the search path, with highest sensitivity at 0–5 m), supporting the use of search grid spacing of less than 10 m in field deployments, and in visually complex environments, outperformed trained human searchers. These results highlight the value of participatory detection dog programs as a scalable and complementary tool for invasive species surveillance. When integrated with traditional visual surveys, trained canine teams can enhance early detection capacity, particularly in habitats where cryptic targets are difficult to locate visually. As invasive species management increasingly relies on rapid, field-deployable detection methods, community-based detection dog programs represent a promising and operationally relevant approach.

## Supplemental Information

10.7717/peerj.21387/supp-1 Supplemental Information 1 Author Arrive 2.0 checklist

## Additional Information and Declarations

### Field Study Permissions

The following information was supplied relating to field study approvals (i.e., approving body and any reference numbers): Virginia Tech IACUC (VT IACUC No. #23-025) Virginia Tech’s Animal Welfare Assurance Number: D16-00131(A-3208-01) (exp. July 31, 2029) United States Department of Agriculture - Animal and Plant Health Inspection Service - Registration Certificate Number: 52-R-0012.

### Data Availability

The following information was supplied regarding data availability: The data is available through the Virginia Tech Data Repository: Dickinson, Sally; Feuerbacher, Erica; Nita, Mizuho; Aviles-Rosa, Edgar O.; Hall, Nathaniel (2026). Range of Detection. University Libraries, Virginia Tech. Dataset. https://doi.org/10.7294/30984718.v1.

## References

[ref-1] Aviles-Rosa EO, Kane SA, Nita M, Feuerbacher E, Hall NJ (2023). Olfactory threshold of dogs (*Canis lupus familiaris*) to cold-killed spotted lantern fly eggs. Applied Animal Behaviour Science.

[ref-2] Bates D, Mächler M, Bolker B, Walker S (2015). Fitting linear mixed-effects models using lme4. Journal of Statistical Software.

[ref-3] Beebe SC, Howell TJ, Bennett PC (2016). Using scent detection dogs in conservation settings: a review of scientific literature regarding their selection. Frontiers in Veterinary Science.

[ref-4] Bennett EM, Hauser CE, Moore JL (2020). Evaluating conservation dogs in the search for rare species. Conservation Biology.

[ref-5] Bullock R, Moylett H, Para G (2017). Technical working group summary report spotted lanternfly, *Lycorma delicatula*. Technical report.

[ref-6] Cristescu RH, Foley E, Markula A, Jackson G, Jones D, Frère C (2015). Accuracy and efficiency of detection dogs: a powerful new tool for koala conservation and management. Scientific Reports.

[ref-7] Dara SK, Barringer L, Arthurs SP (2015). *Lycorma delicatula* (Hemiptera: Fulgoridae): a new invasive pest in the United States. Journal of Integrated Pest Management.

[ref-8] Dickinson S, Nita M, Aviles-Rosa EO, Hall N, Feuerbacher EN (2025). Evaluating the effectiveness of participatory science dog teams to detect devitalized Spotted Lanternfly (*Lycorma delicatula*) egg masses. PeerJ.

[ref-9] Essler JL, Kane SA, Collins A, Ryder K, DeAngelo A, Kaynaroglu P, Otto CM (2021). Egg masses as training aids for spotted lanternfly *Lycorma delicatula* detection dogs. PLOS ONE.

[ref-10] Fuller AK, Augustine BC, Clifton EH, Hajek AE, Blumenthal A, Beese J, Hurt A, Brown-Lima CJ (2024). Effectiveness of canine-assisted surveillance and human searches for early detection of invasive spotted lanternfly. Ecosphere.

[ref-11] Glen AS, Veltman CJ (2018). Search strategies for conservation detection dogs. Wildlife Biology.

[ref-12] Hall NJ, Glenn K, Smith DW, Wynne CDL (2015). Performance of Pugs, German Shepherds, and Greyhounds (*Canis lupus familiaris*) on an odor-discrimination task. Journal of Comparative Psychology.

[ref-13] Keller JA, Hoover K (2023). Approach to surveying egg masses of the invasive spotted lanternfly (Hemiptera: Fulgoridae). Environmental Entomology.

[ref-14] McKeague B, Finlay C, Rooney N (2024). Conservation detection dogs: a critical review of efficacy and methodology. Ecology and Evolution.

[ref-15] R Core Team (2025). R: a language and environment for statistical computing. https://www.R-project.org/.

[ref-16] Rutter NJ, Howell TJ, Stukas AA, Pascoe JH, Bennett PC (2021). Can volunteers train their pet dogs to detect a novel odor in a controlled environment in under 12 weeks?. Journal of Veterinary Behavior.

[ref-17] Rutter NJ, Stukas AA, Howell TJ, Pascoe JH, Bennett PC (2022). Improving access to conservation detection dogs: identifying motivations and understanding satisfaction in volunteer handlers. Wildlife Research.

[ref-18] Urban JM (2020). Perspective: shedding light on spotted lanternfly impacts in the USA. Pest Management Science.

[ref-19] Wickens TD (2002). Elementary signal detection theory.

[ref-20] Wohlfahrt G, Schmitt M, Zeller L, Hörand A, Spittel-Schnell K, Wulms T, Schnell R, Bültge M (2023). Air temperature and humidity effects on the performance of conservation detection dogs. Applied Animal Behaviour Science.

